# Discovery of a Small-Molecule Inhibitor Targeting the ELF3-HSP27 Interaction to Suppress Breast Cancer Progression

**DOI:** 10.3390/ph19050739

**Published:** 2026-05-08

**Authors:** Yi Liu, Sehyun Jung, Soo-Yeon Hwang, Hyunji Jo, Yunjee Bang, Yuna Lee, Jae-Ho Shin, Younghwa Na, Youngjoo Kwon

**Affiliations:** 1 Graduate School of Pharmaceutical Sciences, College of Pharmacy, Ewha Womans University, Seoul 03760, Republic of Korea; liuyi980216@gmail.com (Y.L.); sehyunnni@naver.com (S.J.); hilarious06@naver.com (S.-Y.H.); angella502@naver.com (H.J.); yjbang07@naver.com (Y.B.); dldbsk3321@gmail.com (Y.L.); 2 Graduate Program in Innovative Biomaterials Convergence, Ewha Womans University, Seoul 03760, Republic of Korea; 3 College of Pharmacy, CHA University, Pocheon 11160, Republic of Korea; wogh975@naver.com

**Keywords:** ELF3, HSP27, breast cancer, protein–protein interaction

## Abstract

**Background:** Breast cancer remains a leading cause of cancer-related mortality in women, largely due to metastasis and treatment resistance. ELF3, an ETS transcription factor, has been linked to cancer progression; however, the mechanisms regulating its activity remain incompletely understood. **Methods:** ELF3 expression and its association with patient survival were analyzed using GEO datasets and the Kaplan–Meier Plotter platform. Functional studies were performed using ELF3 knockdown in breast cancer cell lines, followed by WST-1 assays and crystal violet staining. Protein–protein interactions were evaluated using co-expression analysis, immunofluorescence, split luciferase complementation, GST pull-down, and yeast two-hybrid assays. Cycloheximide chase assays were conducted to assess ELF3 protein stability. A panel of small molecules was screened to identify inhibitors of the ELF3-HSP27 interaction, and a lead compound was further validated using biochemical and functional assays. Antitumor activity was evaluated in a xenograft mouse model. **Results:** High ELF3 expression was associated with poorer overall survival in breast cancer patients. HSP27 was identified as a binding partner that stabilizes ELF3 protein, thereby promoting breast cancer cell proliferation. A novel small-molecule inhibitor disrupting the ELF3-HSP27 interaction suppressed cancer cell growth in vitro and reduced tumor growth in vivo. **Conclusions:** The ELF3-HSP27 interaction represents a previously unrecognized contributor to breast cancer progression, and its disruption provides a promising therapeutic strategy.

## 1. Introduction

Cancer remains a significant contributor to global mortality and continues to pose a considerable challenge to increasing life expectancy worldwide. Breast cancer is the most frequently diagnosed malignancy and the leading cause of cancer-related death among women [1]. Notably, the majority of breast cancer-associated deaths are attributed to recurrence and metastasis. Although early-stage diagnosis is generally associated with a favorable prognosis, invasive breast cancer is characterized by aggressive progression and reduced survival rates [2,3].

The ETS transcription factor family has attracted considerable attention due to its critical roles in cancer development and progression [4]. This family comprises approximately 30 members sharing a conserved ETS DNA-binding domain. E74-like ETS transcription factor 3 (ELF3), also known as *ESX*, *ESE-1*, *ERT*, or *jen*, belongs to the ELF subfamily and is predominantly expressed in epithelial tissues [5]. ELF3 exhibits context-dependent roles in tumorigenesis, functioning either as a tumor suppressor or an oncogene depending on the cancer type. For instance, ELF3 suppresses gallbladder cancer progression [6], whereas elevated ELF3 expression is associated with poor prognosis in lung cancer [7,8] and promotes malignant transformation of mammary epithelial cells, enhancing migration, invasion, and anchorage-independent growth in breast cancer [9,10].

Heat shock proteins (HSPs) are a highly conserved family of stress-responsive molecular chaperones that regulate protein folding, stability, and proteostasis through interactions with specific client proteins [11]. Among them, HSP27, HSP60, HSP70, and HSP90 are well-characterized chaperones involved in maintaining protein homeostasis and regulating diverse signaling pathways [12,13]. In particular, HSP27 plays multifaceted roles in cellular processes, including inhibition of protein aggregation, modulation of apoptotic signaling, regulation of intracellular redox balance, and stabilization of cytoskeletal dynamics [14]. Importantly, accumulating evidence has implicated HSP27 in cancer progression, where it enhances tumorigenicity, promotes resistance to anticancer therapies, and suppresses apoptosis [15,16,17,18,19].

Emerging studies suggested that the biological functions of ELF3 are largely dependent on its interacting partners. ELF3 interacts with MED23 to regulate HER2 expression, thereby modulating tumor cell survival, proliferation, and differentiation [20]. In addition, ELF3 forms a protein–protein interaction with SET8 to regulate MARK4 expression, contributing to hyperglycaemia-mediated endothelial NLRP3 inflammasome activation [21]. Despite these findings, whether molecular chaperones such as HSP27 interact with ELF3 to regulate its stability and transcriptional activity in cancer progression remains largely unexplored.

In this study, we investigated the molecular relationship between ELF3 and HSP27 in breast cancer. We demonstrated a direct protein–protein interaction between ELF3 and HSP27 within the transcriptional activation domain (TAD) of ELF3. Mechanistically, HSP27 stabilizes ELF3 protein and promotes its nuclear localization, thereby enhancing its transcriptional activity. Furthermore, our findings suggest that disruption of the ELF3-HSP27 interaction represents a potential therapeutic strategy for breast cancer.

## 2. Results

### 2.1. ELF3 Promotes Breast Cancer Cell Proliferation

**Figure 1:**
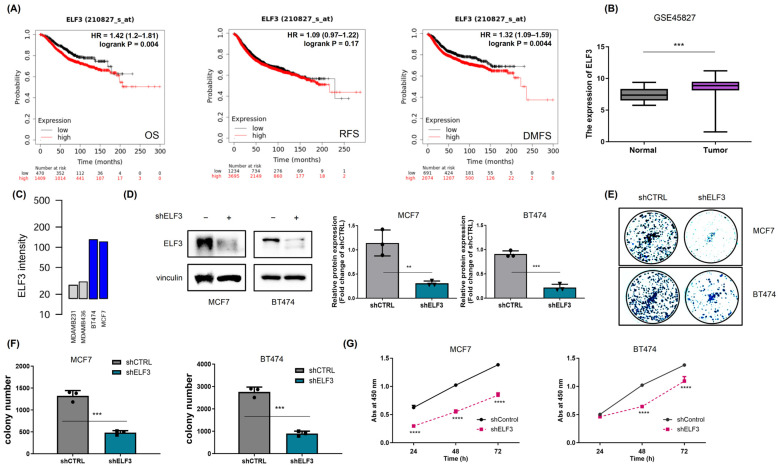
ELF3 promotes breast cancer cell proliferation. (**A**) Kaplan–Meier survival analysis of overall survival (OS), relapse-free survival (RFS), and distant metastasis-free survival (DMFS) in breast cancer patients stratified by ELF3 expression (High vs. Low). (**B**) ELF3 expression levels in normal breast tissues (*n* = 11) and breast cancer tissues (*n* = 130) from the GSE45827 dataset. (**C**) Relative ELF3 expression levels in MDA-MB-231, MDA-MB-436, MCF7, and BT474 cell lines obtained from the GOBO database. Gray bars indicate TNBC cell lines (MDA-MB-231 and MDA-MB-436), whereas blue bars indicate luminal breast cancer cell lines (MCF7 and BT474). (**D**) Validation of ELF3 knockdown in MCF7 and BT474 cells. (**E**,**F**) Long-term cell proliferation assessed by crystal violet staining following ELF3 knockdown. (**G**) Short-term cell viability measured by WST-1 assay. Data are presented as mean ± SD (*n* = 3 independent experiments). Statistical significance was determined using Student’s *t*-test (two groups) or two-way ANOVA (multiple groups). ** *p* < 0.01, *** *p* < 0.001, **** *p* < 0.0001. Circles, squares, and triangles indicate different experimental groups, and each symbol represents an independent biological replicate.

Previous studies have demonstrated that increased ELF3 expression is associated with metastasis, enhanced cell growth, and poor prognosis across multiple cancer types [7,8,9,10]. Thus, to assess the prognostic significance of ELF3 in breast cancer, we analyzed datasets from the Kaplan–Meier Plotter (https://kmplot.com/analysis/ (accessed on 31 May 2023)). High ELF3 expression was significantly correlated with poorer overall survival and distant metastasis-free survival in breast cancer patients (Figure 1A). We next investigated ELF3 expression levels in breast cancer patients and found that ELF3 expression was significantly higher in tumor tissues than in normal breast tissues (Figure 1B). To identify appropriate in vitro models, we analyzed the Gene Expression-Based Outcome for Breast Cancer Online (GOBO) database and found that the MCF7 and BT474 cell lines exhibit relatively high and comparable levels of ELF3 expression (Figure 1C). Based on these findings, ELF3 knockdown was performed in these two cell lines (Figure 1D). Silencing of ELF3 resulted in a significant reduction in cell proliferation, as assessed by proliferation assays (Figure 1E–G). Collectively, these results support a functional role for ELF3 in promoting breast cancer cell proliferation.

### 2.2. ELF3 Positively Correlates with and Directly Interacts with HSP27 in Breast Cancer

**Figure 2:**
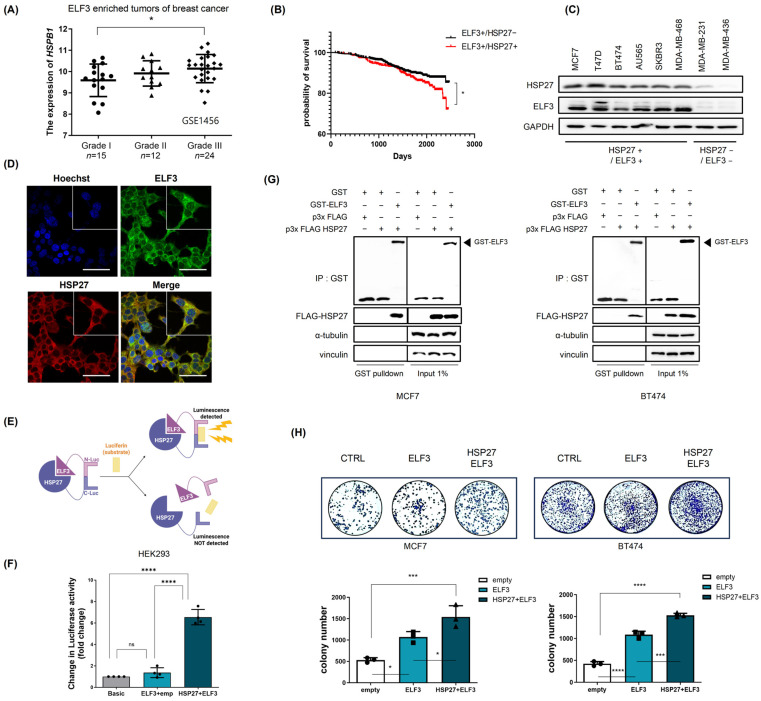
ELF3 positively correlates with and directly interacts with HSP27 in breast cancer. (**A**) *HSPB1* (HSP27) expression according to tumor grade in luminal breast cancer samples with high ELF3 expression (stage I, *n* = 15; stage II, *n* = 12; stage III, *n* = 24). (**B**) Survival analysis comparing breast cancer patients with high ELF3 expression alone and those with concurrent high expression of *ELF3* and *HSPB1* (GSE96058, *n* = 1212). (**C**) Protein expression levels of ELF3 and HSP27 in multiple breast cancer cell lines were analyzed by Western blotting. (**D**) Immunofluorescence analysis in BT474 cells showing the cytoplasmic localization and co-localization of ELF3 (green) and HSP27 (red). Nuclei were stained with DAPI (blue). Scale bar, 50 μm. (**E**) Schematic illustration of the split luciferase complementation assay using N-terminal luciferase-tagged ELF3 (N-luc-ELF3) and C-terminal luciferase-tagged HSP27 (C-luc-HSP27). Created in BioRender. Y.L. (2026) https://app.biorender.com/illustrations/69ce266e40b06e9c0d782942. (**F**) Luciferase activity in HEK293 cells transfected with empty vector, ELF3 alone, or ELF3 and HSP27 expression vectors (*n* = 4). (**G**) Interaction between ELF3 and HSP27 validated by GST pull-down assays followed by Western blotting in MCF7 and BT474 cells. (**H**) Clonogenic assays showing enhanced cell growth upon co-expression of ELF3 and HSP27 in MCF7 and BT474 cells (*n* = 3). Data are presented as mean ± SD. Statistical significance was determined using one-way ANOVA. * *p* < 0.05, *** *p* < 0.001, **** *p* < 0.0001, ns indicated no statistical significance. Circles, squares, and triangles indicate different experimental groups, and each symbol represents an independent biological replicate.

Analysis of public datasets revealed that among HSP family members, *HSPB1* (encoding HSP27) expression was significantly upregulated with increasing tumor grade in breast cancer patients with high ELF3 expression (Figure 2A). Moreover, survival analysis demonstrated that patients with co-amplification of ELF3 and HSP27 exhibited significantly poorer prognosis compared with those without co-amplification (Figure 2B).

To further investigate the relationship between ELF3 and HSP27, we examined their expression across multiple breast cancer cell lines. ELF3 and HSP27 expression levels showed a positive correlation (Figure 2C). Immunofluorescence analysis revealed that both proteins were predominantly localized in the cytoplasm and exhibited substantial co-localization (Figure 2D), suggesting a potential spatial association. To determine whether ELF3 directly interacts with HSP27, we performed split luciferase complementation assays using C-terminal luciferase-tagged HSP27 (C-luc-HSP27) and N-terminal luciferase-tagged ELF3 (N-luc-ELF3) constructs. Co-expression of ELF3 and HSP27 resulted in a significant increase in luminescence signal (Figure 2E,F), indicating a direct physical interaction between ELF3 and HSP27 proteins. This interaction was further validated by GST pull-down assays (Figure 2G). Functionally, co-expression of ELF3 and HSP27 significantly enhanced breast cancer cell proliferation compared with ELF3 expression alone (Figure 2H), suggesting that HSP27 may augment ELF3-mediated tumor cell growth.

### 2.3. HSP27 Regulates ELF3 Protein Stability and Subcellular Localization

**Figure 3:**
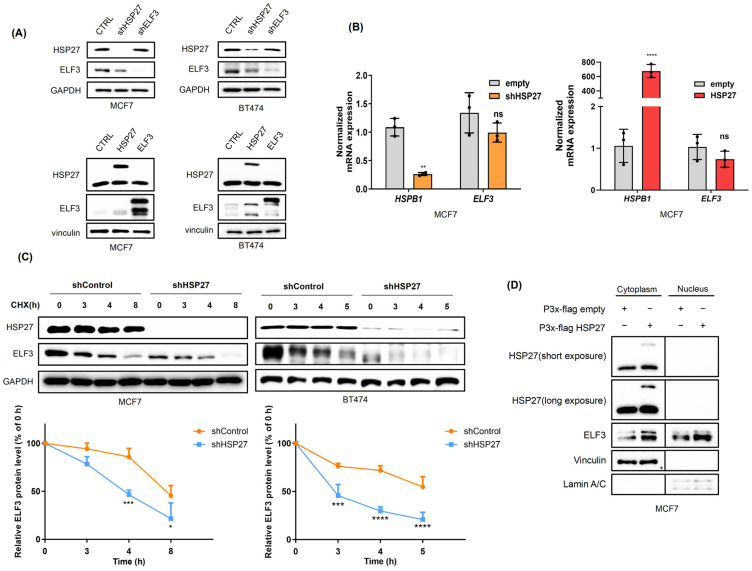
HSP27 regulates ELF3 protein stability and subcellular localization. (**A**,**B**) MCF7 and BT474 cells were subjected to HSP27 or ELF3 knockdown or overexpression. Protein and mRNA levels were analyzed by Western blotting (**A**) and qRT-PCR (**B**) at 24 h post-transfection. (**C**) Cycloheximide (CHX; 5 μg/mL) chase assay in MCF7 and BT474 cells with shCTRL or shHSP27, showing ELF3 protein stability over time. Data are presented as mean ± SD (*n* = 3 independent experiments). Statistical significance was determined using two-way ANOVA. * *p* < 0.05, ** *p* < 0.01, *** *p* < 0.001, **** *p* < 0.0001, ns indicated no statistical significance. Circles, squares, and triangles indicate different experimental groups, and each symbol represents an independent biological replicate. (**D**) Subcellular fractionation followed by Western blotting showing changes in ELF3 localization upon HSP27 modulation. Lamin A/C and GAPDH were used as nuclear and cytoplasmic markers, respectively.

To clarify the regulatory relationship between ELF3 and HSP27, gain- and loss-of-function experiments were performed in MCF7 and BT474 cells. Silencing HSP27 resulted in a marked reduction in ELF3 protein levels, whereas HSP27 overexpression significantly increased ELF3 protein abundance. In contrast, modulation of ELF3 expression did not affect HSP27 protein levels (Figure 3A). Notably, changes in HSP27 expression did not alter ELF3 mRNA levels (Figure 3B), indicating that HSP27 regulates ELF3 at the post-transcriptional level, likely through modulation of protein stability.

To directly assess ELF3 protein stability, cycloheximide (CHX) chase assays were performed. Following CHX treatment, ELF3 protein levels declined more rapidly in HSP27-silenced cells compared with control cells, indicating reduced ELF3 protein stability (Figure 3C). Consistently, the calculated half-life of ELF3 was 7.43 ± 0.99 h in MCF7 shControl cells and decreased to 4.03 ± 0.34 h in shHSP27 cells. Similarly, in BT474 cells, the half-life of ELF3 was greater than 5 h in shControl cells but was markedly reduced to 1.65 ± 0.84 h upon HSP27 silencing. Together with the GST pull-down results (Figure 2G), these findings demonstrate that HSP27 stabilizes ELF3 protein through direct binding.

Given that ELF3 functions as a transcription factor requiring nuclear localization, we next examined whether HSP27 affects the subcellular distribution of ELF3. HSP27 overexpression enhanced ELF3 protein levels and promoted its nuclear translocation (Figure 3D). Collectively, these results indicate that HSP27 acts as a molecular chaperone that stabilizes ELF3 and facilitates its nuclear localization, thereby supporting its transcriptional activity.

### 2.4. HSP27 Binds to the Transactivation Domain (TAD) of ELF3

**Figure 4:**
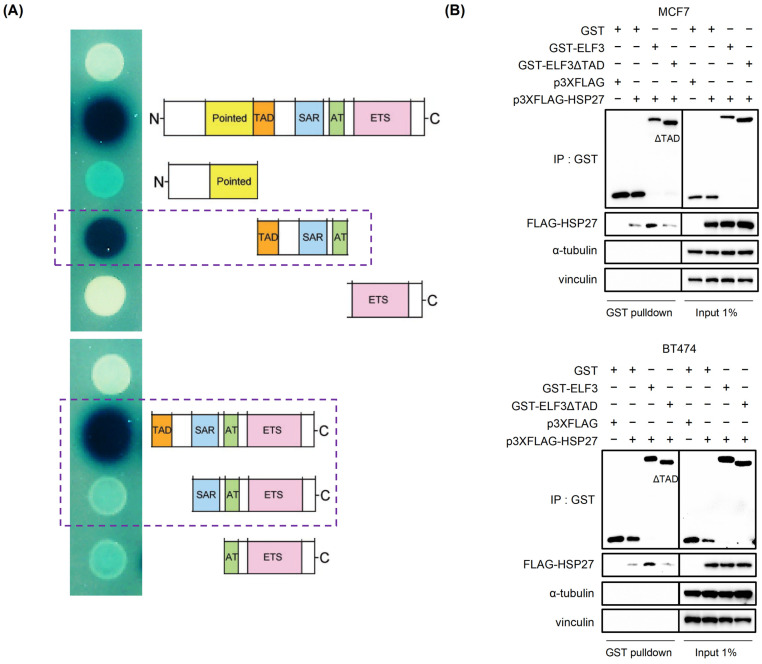
HSP27 binds to the transactivation domain (TAD) of ELF3. (**A**) Yeast two-hybrid assay assessing the interaction between HSP27 and full-length ELF3 or ELF3 truncation/deletion mutants containing different domain combinations (PNT, TAD, SAR, AT, and ETS). The upper dashed box highlights the interaction between ELF3 and HSP27 observed only in the presence of the TAD of ELF3, whereas the lower dashed box indicates the altered interaction pattern observed in the absence of the TAD. (**B**) GST pull-down assay examining the interaction between HSP27 and GST-tagged full-length ELF3 (GST-ELF3) or TAD-deleted ELF3 (GST-ELF3ΔTAD). MCF7 and BT474 cells were co-transfected with FLAG-HSP27 and the indicated GST constructs for 24 h.

Previous studies have shown that the transactivation domain (TAD) of ELF3 serves as a key interface for coactivator interactions [22,23]. We therefore investigated whether the interaction between HSP27 and ELF3 is mediated through TAD of ELF3. A yeast two-hybrid assay was performed to assess protein–protein interactions. As shown in Figure 4A, a strong interaction signal was observed in the presence of the ELF3 TAD, whereas deletion of the TAD domain markedly reduced the interaction. To further validate this finding, a GST-tagged ELF3 construct lacking the TAD domain (ELF3-ΔTAD) was generated and subjected to GST pull-down assays. Consistently, deletion of the TAD domain significantly reduced the binding of ELF3 to HSP27 compared with the full-length protein (Figure 4B). Collectively, these results suggest that the TAD domain of ELF3 is a critical region mediating its interaction with HSP27.

### 2.5. Identification of HT81 as a Small-Molecule Inhibitor of the ELF3-HSP27 Interaction

**Figure 5:**
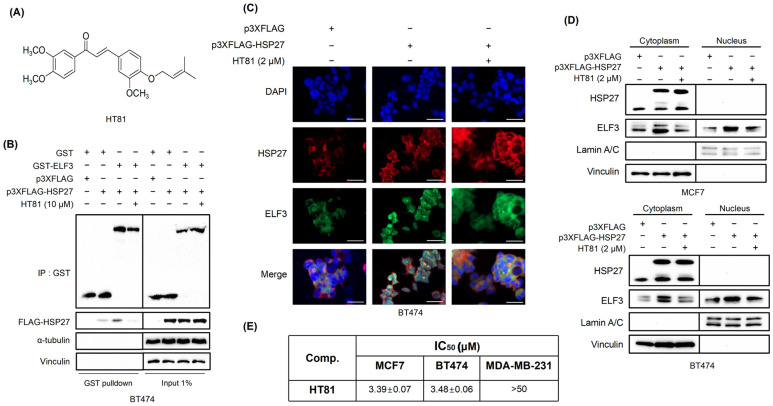
Identification of HT81 as a small-molecule inhibitor of the ELF3-HSP27 interaction. (**A**) Chemical structure of HT81. (**B**) GST pull-down assay assessing the effect of HT81 on the interaction between ELF3 and HSP27. (**C**,**D**) HT81-mediated changes in ELF3 localization analyzed by subcellular fractionation followed by immunofluorescence staining (**C**) and Western blotting (**D**) in MCF7 and BT474 cells. Scale bar, 50 μm. (**E**) Cell viability of MCF7, BT474, and MDA-MB-231 cells following HT81 treatment for 72 h, measured by WST-1 assay. Data are presented as mean ± SD (*n* = 3 independent experiments).

To identify small molecules capable of disrupting the ELF3-HSP27 interaction, we performed a split luciferase-based biosensor screening in HEK293 cells using a panel of 29 newly synthesized chalcone-derived compounds. Among them, HT81 (Figure 5A) showed the strongest inhibitory effect, suppressing the ELF3-HSP27 interaction by more than 70% (Supplementary Figure S1). The inhibitory effect of HT81 was further evaluated using a GST pull-down assay. Treatment with HT81 (10 μM) reduced the binding between ELF3 and HSP27 (Figure 5B), confirming its ability to interfere with their interaction. Given that HSP27 overexpression promotes nuclear translocation of ELF3 (Figure 3D), we examined whether HT81 affects this process. Immunofluorescence analysis showed that HT81 treatment reduced HSP27-induced nuclear accumulation of ELF3 (Figure 5C). Consistently, subcellular fractionation assays demonstrated decreased ELF3 levels in the nuclear fraction following HT81 treatment under conditions of HSP27 overexpression (Figure 5D). Functionally, HT81 exhibited anti-proliferative effects in MCF7 and BT474 cells, which express relatively high levels of both HSP27 and ELF3, whereas MDA-MB-231 cells, with lower expression of these proteins (Figure 2C), showed reduced sensitivity to HT81 (Figure 5E). Collectively, these results suggest that HT81 interferes with the ELF3-HSP27 interaction and modulates ELF3 nuclear localization, leading to reduced breast cancer cell proliferation.

### 2.6. HT81 Exerts Anticancer Efficacy Through Disruption of the ELF3-HSP27 Interaction

**Figure 6:**
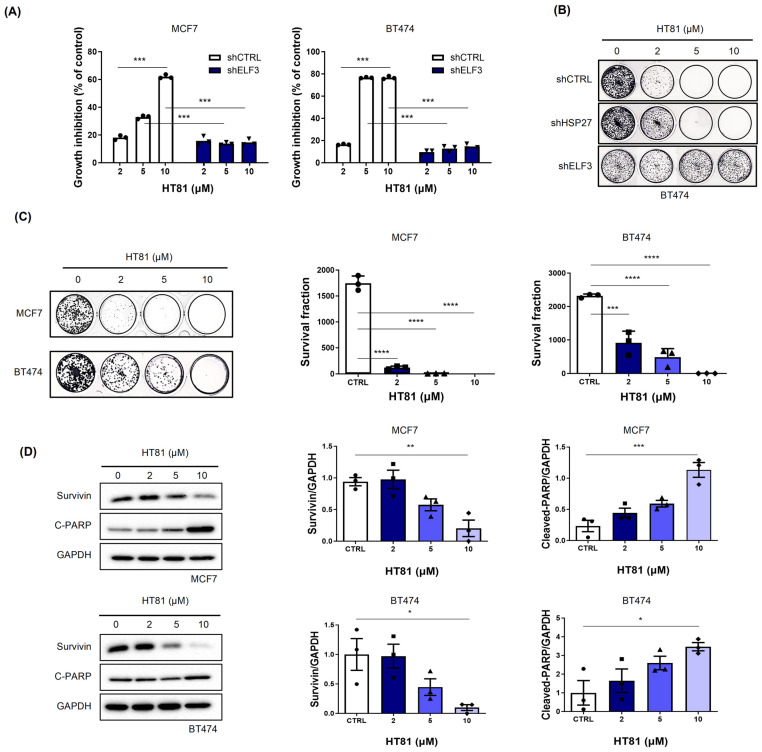
HT81 exerts anticancer effects through disruption of the ELF3-HSP27 interaction. (**A**) Cell viability of control (shCTRL) and ELF3-knockdown (shELF3) MCF7 cells following HT81 treatment, measured by WST-1 assay. (**B**) Clonogenic assays in BT474 cells with control, HSP27 knockdown, or ELF3 knockdown. Cells were treated with HT81 (0–10 μM) for 10 days and stained with 2% crystal violet. (**C**) Clonogenic assays in parental MCF7 and BT474 cells treated with HT81 (0–10 μM) for 10 days followed by crystal violet staining. (**D**) Western blot analysis of apoptotic markers in MCF7 and BT474 cells following HT81 treatment (0–10 μM). Data are presented as mean ± SD (*n* = 3 independent experiments). Statistical significance was determined using one-way ANOVA. * *p* < 0.05, ** *p* < 0.01, *** *p* < 0.001, **** *p* < 0.0001. Circles, squares, and triangles indicate different experimental groups, and each symbol represents an independent biological replicate.

To investigate the mechanism and potential anticancer effects of HT81, we evaluated its activity in ELF3-knockdown (shELF3) and control (shCTRL) cells using WST-1 assays. HT81 showed minimal inhibitory effects on cell viability in ELF3-knockdown cells across the tested concentrations, suggesting that its anti-proliferative activity is dependent on ELF3 (Figure 6A). Although HT81 showed minimal inhibitory effects in ELF3-knockdown cells, a slight decrease in cell viability was observed at 10 μM. This effect was relatively small and not statistically significant, but it may suggest a potential off-target effect at higher concentrations, which warrants further investigation across a broader concentration range.

A 10-day clonogenic assay further demonstrated that HT81 dose-dependently suppressed colony formation in BT474_shCTRL cells, while this effect was attenuated in BT474_shHSP27 cells and largely abolished in BT474_shELF3 cells (Figure 6B). These results indicate that the anti-proliferative effects of HT81 are associated with the ELF3-HSP27 axis. We next tested the effects of HT81 in parental breast cancer cells. HT81 exhibited anti-proliferative activity, with significant growth inhibition observed at 2 μM in both MCF7 and BT474 cells (Figure 6C). Moreover, HT81 induced apoptosis in a dose-dependent manner, as evidenced by decreased expression of the anti-apoptotic protein survivin and increased levels of cleaved PARP (C-PARP) (Figure 6D). Collectively, these findings demonstrate that HT81 suppresses breast cancer cell proliferation and induces apoptosis, at least in part through disruption of the ELF3-HSP27 interaction.

### 2.7. HT81 Suppresses Tumor Growth in a Breast Cancer Xenograft Model

**Figure 7:**
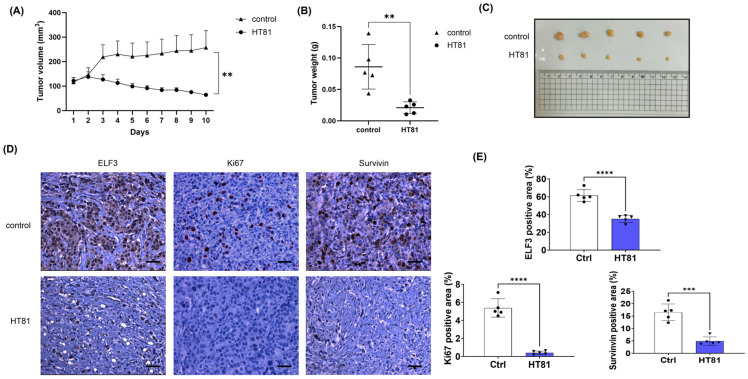
HT81 suppresses tumor growth in a breast cancer xenograft model. (**A**) Tumor growth curves of BT474 xenografts in SCID mice following subcutaneous implantation of BT474 cells (1 × 10^7^ cells per mouse, *n* = 5/group). HT81 was administered intraperitoneally at 20 mg/kg/day once tumor volumes reached approximately 80 mm^3^, and tumor volume was monitored daily thereafter. (**B**) Tumor weights measured at the experimental endpoint. (**C**) Representative images of excised tumors from control and HT81-treated groups. (**D**) Representative immunohistochemical staining of ELF3, Ki-67, and survivin in tumor sections. Nuclei were counterstained with hematoxylin, and protein expression was visualized using DAB. Scale bar, 50 μm. (**E**) Quantification of ELF3-, Ki-67-, and survivin-positive staining in tumor tissues. Data are presented as mean ± SD. Statistical significance was determined using Student’s *t*-test. ** *p* < 0.01, *** *p* < 0.001, **** *p* < 0.0001.

A BT474 xenograft model was established in SCID mice to evaluate the antitumor efficacy of HT81. HT81 was administered daily at 20 mg/kg/day for 10 consecutive days. Tumor growth was monitored over time, and a significant reduction in tumor volume and weight was observed in the HT81-treated group compared with controls (Figure 7A–C). Immunohistochemical analysis further demonstrated that HT81 treatment reduced ELF3 expression in tumor tissues. In addition, the proliferation marker Ki-67 and the anti-apoptotic marker survivin were significantly decreased in the HT81-treated tumors compared with controls (Figure 7D,E). Collectively, these results suggest that HT81 suppresses tumor growth in vivo, potentially through disruption of ELF3-HSP27 interaction, leading to reduced ELF3 expression and increased apoptosis.

## 3. Discussion

In this study, we demonstrated that ELF3 expression is significantly elevated in breast cancer tissues and is associated with poor patient survival. Functional analyses further showed that ELF3 promotes breast cancer cell proliferation, supporting its role as a potential oncogenic driver in this context.

ELF3 is a member of the ETS transcription factor family and exhibits context-dependent functions in tumorigenesis, acting either as an oncogene or a tumor suppressor depending on the cancer type [5,6,7,8,9,10]. While ELF3 has been implicated in promoting tumor progression in lung, breast, and liver cancers, it has also been reported to suppress tumor growth in certain malignancies, such as cholangiocarcinoma [24]. Additionally, ELF3 has been shown to regulate invasion and epithelial–mesenchymal transition (EMT) in a context-dependent manner [25]. These findings highlight the complex and multifaceted role of ELF3 in cancer biology. However, the precise role of ELF3 in breast cancer remains incompletely understood.

Our previous studies demonstrated that ELF3 interacts with MED23 to regulate HER2 transcription, and disruption of the ELF3-MED23 interaction suppresses HER2-driven tumorigenesis [20]. Building on this concept, we hypothesized that additional protein–protein interactions may regulate ELF3 function. In the present study, we identified HSP27 as a novel binding partner of ELF3 and showed that HSP27 stabilizes ELF3 protein and promotes its nuclear localization.

Mechanistically, we confirmed a direct interaction between HSP27 and ELF3 using split luciferase and GST pull-down assays. Although ELF3 is primarily recognized as a nuclear transcription factor, our data indicated that ELF3 is also present in the cytoplasm, where its interaction with HSP27 may regulate protein stability prior to nuclear translocation.

Heat shock proteins (HSPs) have received attention for their role as chaperones involved in interactions with various proteins [26]. Overexpression of HSPs is associated with poor prognosis in certain types of cancers, including survival rates and treatment responses [27,28,29,30,31]. HSP27 has been reported to interact with high-mobility group nucleosome-binding domain 5 (HMGN5), thereby facilitating the activation of tumorigenesis-related signaling pathways [32]. In esophageal cancer stem cells, HSP27 directly links AKT to the mTOR pathway, leading to increased HK2 expression and enhanced metabolic reprogramming [33]. Furthermore, HSP27 has been reported to interact with prolyl 4-hydroxylase subunit alpha 2 (P4HA2), promoting EGFR phosphorylation and activating the EGFR/ERK signaling pathway, thereby driving glioma progression [34]. Consistent with these findings, our study identifies ELF3 as a novel HSP27 client protein and further supports the role of HSP27 as a regulatory hub that modulates multiple cancer-related signaling pathways.

Although we demonstrated that the ELF3–HSP27 interaction promotes ELF3 nuclear localization and contributes to enhanced breast cancer cell proliferation and reduced apoptosis, the underlying molecular mechanisms and specific downstream transcriptional effectors remain to be fully elucidated. Previous studies have reported that ELF3 can promote cancer cell proliferation by activating multiple oncogenic pathways, including β-catenin, PI3K/AKT, IGF, VEGF, and HIF-α signaling [5]. Therefore, further investigations are required to determine whether these pathways are involved in ELF3–HSP27-mediated breast cancer progression and to identify additional downstream regulatory mechanisms.

Protein–protein interactions (PPIs) play essential roles in numerous biological processes, and their dysregulation contributes to the development of various diseases, including cancer [35]. Accordingly, targeting PPIs has emerged as a promising therapeutic strategy for anticancer drug development. Several studies have demonstrated the feasibility of PPI inhibition in cancer [36,37,38]. For example, IAG933 disrupts the YAP-TEAD interaction, thereby reducing lung cancer cell proliferation and tumorigenesis [39]. Similarly, small-molecule inhibitors such as 18β-glycyrrhetinic acid derivatives disrupt the Hsp90-Cdc37 interaction, leading to reduced proliferation and migration, induction of apoptosis, and cell cycle arrest in lung cancer cells [40]. Notably, PPI-targeting approaches enable selective modulation of tumor-specific signaling networks, potentially minimizing systemic toxicity [41]. Collectively, these findings support the therapeutic potential of PPI-targeting approaches and provide a rationale for developing inhibitors that disrupt oncogenic protein complexes.

In this study, we identified HT81 as a potent small-molecule compound capable of interfering with the ELF3-HSP27 interaction. Functional analyses demonstrated that HT81 inhibits breast cancer cell proliferation, particularly under conditions of elevated ELF3 and HSP27 expression. In addition, HT81 exhibited antitumor activity in a breast cancer xenograft model, supporting its potential therapeutic relevance.

The compound screening was performed using a focused series of 29 newly synthesized chalcone-derived compounds. This scaffold was selected because chalcone derivatives are structurally flexible and have been widely explored in the development of small molecules targeting protein–protein interactions. However, the relatively small size of the screening library limits the generalizability of the structure–activity relationship. Although HT81 suppressed the ELF3-HSP27 interaction and showed anti-proliferative and antitumor effects, several limitations should be acknowledged. HT81 should therefore be considered a preliminary lead compound for further optimization. The direct binding mode of HT81 has not yet been determined, and additional target-engagement assays are needed to clarify whether HT81 directly binds ELF3, HSP27, or their interaction interface. Moreover, broader chemical library screening and systematic structural optimization will be necessary to improve potency, selectivity, and drug-like properties. In addition, the downstream transcriptional programs regulated by the ELF3-HSP27 axis, as well as potential off-target effects of HT81, remain to be elucidated in future studies. Structural modeling and docking analyses will be valuable for defining the ELF3-HSP27 interaction interface and for providing mechanistic insight into HT81-mediated disruption. Such studies may also guide future optimization of HT81 and related analogs.

Notably, HT81 showed limited growth-inhibitory activity in MDA-MB-231 cells, which may be related to the relatively low expression of ELF3 and HSP27. However, because MDA-MB-231 is a triple-negative breast cancer cell line, the lack of response may not be solely explained by target expression levels. TNBC cells may rely on distinct oncogenic drivers, which could make the ELF3-HSP27 axis less critical in this context. Further evaluation of HT81 in additional TNBC models, particularly those with high ELF3 and HSP27 expression, will be necessary to clarify this issue.

Overall, our findings demonstrate that ELF3 is associated with poor prognosis in breast cancer and is positively correlated with HSP27 expression. Mechanistically, HSP27 interacts with the TAD of ELF3 in the cytoplasm, stabilizing ELF3 protein and facilitating its nuclear localization. These findings suggest that HSP27 regulates ELF3 primarily at the protein level and contributes to its functional activity as a transcription factor. Importantly, targeting the ELF3-HSP27 interaction represents a promising strategy for inhibiting ELF3-driven tumor progression. Further studies are warranted to elucidate the detailed molecular mechanisms and to evaluate the clinical potential of disrupting this interaction.

## 4. Materials and Methods

### 4.1. HT81 Synthesis

HT81 was synthesized and achieved a purity of 99.9%, confirmed by high-performance liquid chromatography (HPLC). Detailed information regarding the synthesis process along with the ^1^H and ^13^C nuclear magnetic resonance spectral data, HPLC chromatograms, and the liquid chromatography–high-resolution mass spectroscopic data for HT81 can be found in the Supplementary Information (Synthesis of HT81, Figures S2 and S3).

### 4.2. Bioinformatics Analysis

Gene expression datasets (GSE45827 [42], and GSE96058 [43]) were obtained from the Gene Expression Omnibus. GSE45827 was used to evaluate ELF3 expression levels in breast cancer tissues. Survival outcomes associated with co-expression of ELF3 and HSPB1 were assessed using GSE96058. Kaplan–Meier survival analysis was performed using the Kaplan–Meier Plotter (https://kmplot.com/analysis/ (accessed on 31 May 2023)).

### 4.3. Cell Culture and Transfection

Human breast cancer cell lines MCF7, T47D, BT474, AU565, SKBR3, MDA-MB-231 and MDA-MB-436 were cultured in RPMI 1640 medium (Welgene, Gyeongsan-si, Gyeongsangbuk-do, Republic of Korea), and the human embryonic kidney cell line HEK293T and 293FT and human breast cancer cell lines MDA-MB-468 were grown in Dulbecco’s modified Eagle’s medium (DMEM; Welgene, Gyeongsan-si, Gyeongsangbuk-do, Republic of Korea). All cell lines were obtained from the Korean Cell Line Bank (KCLB, Seoul, Republic of Korea). All media were supplemented with 10% fetal bovine serum (FBS) and 1% penicillin/streptomycin. Cells were incubated at 37 °C in a humidified atmosphere containing 5% CO_2_. For transfection, plasmid DNA was introduced using JetPRIME^®^ reagent according to the manufacturer’s instructions. Cells were incubated for 24 h following transfection before further analysis.

### 4.4. shRNA-Mediated Knockdown of HSP27 and ELF3

Lentiviral particles were produced in 293FT cells. Cells were seeded in 100 mm dishes and transfected at about 50% confluence with shCTRL (pLKO.1 empty vector), shELF3 (TRCN0000013865, Merck, Darmstadt, Germany), or shHSP27 (sc-29350-SH, Santa Cruz, CA, USA), along with packaging plasmids (pRSV-Rev, pCMV-VSVG, and pMDLg/pRRE), using JetPRIME^®^. After 72 h, viral supernatants were collected, centrifuged at 2000 rpm for 5 min, and filtered through a 0.45 μm polyvinylidene fluoride (PVDF) membrane (Sartorius, Göttingen, Germany). MCF7 and BT474 cells were infected with viral supernatants in the presence of polybrene (8 μg/mL). After 48 h, cells were selected with puromycin (3 μg/mL) for 2 weeks to establish stable cell lines.

### 4.5. Cell Viability (WST-1) Assay

Cells were seeded in 96-well plates at a density of 1 × 10^4^ cells per well. After 4 h of serum starvation, cells were treated with serial dilutions of compounds in serum-free medium. At the indicated time points, 5 μL of EZ-CytoX (DoGenBio Co., Ltd., Seoul, Republic of Korea) was added to each well and incubated for 3 h. Absorbance was measured at 450 nm using a microplate reader (VersaMax, Molecular Devices, San Jose, CA, USA).

### 4.6. Clonogenic Assay

Cells were seeded in a 6-well plate at a density of 5 × 10^3^ cells per well. Media were replaced every two days. After 10 days, colonies were fixed with 100% methanol for 1 h and stained with 2% (*w*/*v*) crystal violet (SAMCHUN Pure Chemical Co., Ltd., Pyeongtaek-si, Gyeonggi-do, Republic of Korea) in methanol. Plates were washed with distilled water and air-dried prior to imaging.

### 4.7. Real-Time qPCR (RT-qPCR)

Total RNA was extracted using Tri-RNA reagent (FAVORGEN Biotech Crop., Pingtung, Taiwan), and complementary DNA (cDNA) was synthesized using the PrimeScript^TM^ RT reagent kit (Takara Bio Inc., Shiga, Japan). Quantitative PCR was performed using the SensiFAST^TM^ SYBR No-ROX kit (Bioline, London, UK) on a CFX96 Real-Time PCR system (Bio-Rad Laboratories, Hercules, CA, USA). Thermal cycling conditions were as follows: initial denaturation at 95 °C for 2 min, followed by 31 cycles of 95 °C for 10 s, 57 °C for 10 s, and 72 °C for 20 s. Relative mRNA expression levels were calculated using the ΔΔCt method and normalized to GAPDH. Primer sequences are listed in Supplementary Information Table S1.

### 4.8. Split Luciferase Biosensor Assay

Split luciferase biosensors were generated from the firefly luciferase gene derived from the pGL3-basic vector (Promega, Madison, WI, USA). The luciferase gene was divided into N-terminal and C-terminal fragments and fused to ELF3 or HSP27 using the In-Fusion^®^ HD Cloning Kit (Takara Bio Inc., Shiga, Japan), generating Nluc-ELF3 and Cluc-HSP27 constructs. HEK293 cells were co-transfected with the indicated plasmids using JetPRIME^®^. After 12 h, cells were treated with HT compounds (10 μM) for an additional 12 h. Luciferase activity was measured using a MicroLumat Plus LB96V luminometer (Berthold GmbH & Co. KG, Bad Wildbad, Germany).

### 4.9. Western Blot Analysis

Cells were lysed in RIPA buffer (Cell Signaling Technology, Danvers, MA, USA) supplemented with 1% protease inhibitor (GenDEPOT, Katy, TX, USA). Lysates were incubated on ice for 10 min and centrifuged at 13,000 rpm for 20 min at 4 °C. The protein concentrations were determined using the Pierce^TM^ BCA Protein Assay Kit (Thermo Fisher Scientific, Waltham, MA, USA). Equal amounts of protein (20 μg) were separated by SDS-PAGE and transferred to a 0.2 μm PVDF membrane (Pall Life Sciences, Port Washington, NY, USA). Membranes were blocked with 5% skim milk for 20 min and incubated overnight at 4 °C with primary antibodies (Supplementary Information Table S2). Protein bands were visualized using the ECL solution reagent (GE Healthcare, Chicago, IL, USA) and detected using LAS-3000 (Fuji Photo Film Co., Ltd., Tokyo, Japan). Densitometric analysis was performed using Image J program (NIH, Bethesda, MD, USA) and normalized to loading controls (GAPDH, vinculin or α-tubulin).

### 4.10. GST Pull-Down Assay

MCF7 and BT474 cells were seeded in 100 mm dishes and transfected with GST-empty, GST-ELF3, and GST-ELF3 ΔTAD constructs, along with p3xFLAG-empty or p3xFLAG-HSP27 using JetPRIME^®^ for 24 h. For compound treatment, cells were treated with HT81 (5 μM) for 12 h. Cells were lysed using NP40 lysis buffer (50 mM Tris-HCl, pH 8, 1% NP40, 150 Mm NaCl, 2 Mm EDTA). Lysates (1 mg total protein) were incubated with glutathione-Sepharose^TM^ 4B beads (Merck, Darmstadt, Germany) overnight at 4 °C. Beads were washed three times with ice-cold 1× PBS and proteins were eluted with glutathione elution buffer (50 mM Tris-HCl pH 8, 10 mM glutathione). Eluted proteins were mixed with 2× loading dye, boiled at 98 °C for 5 min and analyzed by Western blotting.

### 4.11. Immunofluorescence (IF) Assay

BT474 cells were seeded on 8-well chamber slides and fixed with 4% paraformaldehyde for 20 min at room temperature. Cells were permeabilized and blocked with a blocking solution containing 5% Blocking One-P (Nacalai Tesque, Kyoto, Japan) and 0.1% Triton X-100 in PBS. Cells were incubated overnight at 4 °C with primary antibodies against ELF3 and HSP27, followed by incubation with Alexa Fluor^®^ 488 anti-mouse IgG (green) and Alexa Fluor^®^ 568 anti-rabbit IgG (red) secondary antibodies for 1 h at room temperature. Nuclei were stained with DAPI (0.1 μg/mL). Images were acquired using a fluorescence microscope (Apotome, Carl Zeiss Co., Ltd., Jena, Germany) and analyzed with ZEN Pro 2.3 software.

### 4.12. Cycloheximide (CHX) Chase Assay

MCF7 and BT474 cells expressing shCTRL or shHSP27 were treated with cycloheximide (5 μg/mL; Sigma-Aldrich, St. Louis, MO, USA) for the indicated time points. Cells were harvested, and protein levels were analyzed by Western blotting.

### 4.13. Xenograft Mouse Model

Female CB-17 SCID mice (4 weeks old, weighing 18–20 g) were purchased from Koatech (Pyeongtaek, Gyeonggi-do, Republic of Korea). The mice were housed under specific pathogen-free (SPF) conditions in cages with controlled temperature (23 ± 2 °C), humidity (60 ± 2%), and a 12 h light-dark cycle. After one week of acclimatization, BT474 cells (1 × 10^7^) were subcutaneously injected. When tumor volume reached approximately 80 mm^3^, mice were randomized into two groups and treated with saline or HT81 (20 mg/kg/day, intraperitoneally) for 10 days. Tumor volumes were measured daily. After the 10-day treatment period, the mice were euthanized using CO_2_, and tumor tissues were collected for immunohistochemical (IHC) staining. All tumor xenograft experiments were approved by the Institutional Animal Care and Use Committee (IACUC) of Ewha Womans University (Approval Code: EWHA IACUC past-071; Approval Date: 21 January 2021) and conducted in accordance with the ARRIVE guidelines and the National Research Council’s Guide for the Care and Use of Laboratory Animals.

### 4.14. Immunohistochemistry (IHC)

Tumor tissues were fixed, paraffin-embedded, sectioned, and subjected to antigen retrieval. Sections were incubated with primary antibodies (ELF3, Ki-67, survivin), followed by secondary antibodies (Supplementary Information Table S2). Signals were developed using DAB (Dako, CA, USA), and sections were counterstained with hematoxylin. Images were acquired using light microscopy at 200× magnification.

### 4.15. Statistical Analysis

Statistical analysis was performed using GraphPad Prism (Version 10.0.0, GraphPad Software, Inc., San Diego, CA, U.S.A). Data are presented as mean ± SD from at least three independent experiments. Comparisons between two groups were performed using two-tailed unpaired Student’s *t*-tests. Multiple comparisons were analyzed using one-way or two-way ANOVA. A *p*-value < 0.05 was considered statistically significant.

## 5. Conclusions

In summary, our study demonstrates that ELF3 plays a critical role in breast cancer cell proliferation. Mechanistically, ELF3 interacts with HSP27 through its TAD in the cytoplasm, where HSP27 stabilizes ELF3 and promotes its nuclear translocation, thereby supporting its function as a transcription factor. Furthermore, we identified HT81 as a small-molecule inhibitor that interferes with the HSP27-ELF3 interaction and exhibits anticancer activity. These findings suggest that disruption of the HSP27-ELF3 interaction may represent a promising therapeutic strategy for breast cancer.

## Data Availability

The original contributions presented in this study are included in the article and Supplementary Materials. Further inquiries can be directed to the corresponding authors.

## Supplementary Materials

The following supporting information can be downloaded at: https://www.mdpi.com/article/10.3390/ph19050739/s1, Figure S1: Screening of chalcone-derived compounds for inhibition of the ELF3–HSP27 interaction; Figure S2: ^1^H-NMR Spectrum of HT81; Figure S3: ^13^C-NMR Spectrum of HT81; Table S1. PCR primer sequences used in this study; Table S2. Information on antibodies used in this study.

## Abbreviations

The following abbreviations are used in this manuscript:

ANOVA	analysis of variance
BC	breast cancer
C-PARP	cleaved poly (ADP-ribose) polymerase
cDNA	complementary DNA
CHX	cycloheximide
DMEM	Dulbecco’s modified Eagle’s medium
DMFS	distant metastasis-free survival
ELF3	E74-like ETS transcription factor 3
EMT	epithelial–mesenchymal transition
GEO	Gene Expression Omnibus
GOBO	Gene Expression-Based Outcome for Breast Cancer Online
HER2	human epidermal growth factor receptor 2
HMGN5	high-mobility group nucleosome-binding domain 5
HRP	horseradish peroxidase
HSPs	heat shock proteins
IHC	immunohistochemistry
KM plotter	Kaplan–Meier plotter database
MED23	mediator complex subunit 23
NCBI	National Center for Biotechnology Information
OS	overall survival
P4HA2	prolyl 4-hydroxylase subunit alpha 2
PPIs	protein–protein interactions
PVDF	polyvinylidene fluoride
RFS	relapse-free survival
RT-qPCR	real-time quantitative polymerase chain reaction
TAD	transactivation domain
TBS	Tris-buffered saline
TBST	Tris-buffered saline with 0.1% Tween 20
