# Supplementary material for: Discovery of a Small-Molecule Inhibitor Targeting the ELF3-HSP27 Interaction to Suppress Breast Cancer Progression
Source: Pharmaceuticals (Basel). 2026 May 8;19(5):739. doi: 10.3390/ph19050739 (PMC13209529; doi:10.3390/ph19050739)
Supplement: Supplementary file 1 [file pharmaceuticals-19-00739-s001.zip › pharmaceuticals-4292714-supplementary.pdf]

Supplementary information

Discovery of a small-molecule inhibitor targeting the ELF3-HSP27 interaction to suppress breast cancer progression

Yi Liu[1,†], Sehyun Jung[1,†], Soo-Yeon Hwang[1], Hyunji Jo[1], Yunjee Bang[1,2], Yuna Lee[1,2], Younghwa Na[3,*], and Youngjoo Kwon[1,2,*]

[1] College of Pharmacy, Graduate School of Pharmaceutical Sciences, Ewha Womans University, Seoul, 03760, Republic of Korea; liuyi980216@gmail.com (Y.L.), sehyunnni@naver.com (S.J.)
[2] Graduate Program in Innovative Biomaterials Convergence, Ewha Womans University, Seoul, 03760, Republic of Korea
[3] College of Pharmacy, CHA University, Pocheon, 487-010, Republic of Korea; yna7315@cha.ac.kr (Y.N.)
***** Correspondence: ykwon@ewha.ac.kr (Y.K.); Tel.: +82 2-3277-4653; fax: +82 2-3277-3052
[†] These authors contributed equally to this work.

Contents

Synthesis of HT81

Figure S1. Screening of chalcone-derived compounds for inhibition of the ELF3–HSP27 interaction

Figure S2. ^{1}H-NMR Spectrum of **HT81**

Figure S3. ^{13}C-NMR Spectrum of **HT81**

Table S1. PCR primer sequences used in this study

Table S2. Information on antibodies used in this study

Synthesis of HT81

HT 81 was synthesized by utilizing the modified *Claisen-Schmidt* condensation reaction of 3,4-dimethoxy acetophenone and 3-methoxy-4-((3-methylbut-2-en-1-yl)oxy)benzaldehyde in ethanol solvent under NaOH basic conditions. In the ^{1}H-NMR spectrum, trans α,β-unsaturated structure of the chalcone was confirmed by observing a set of doublet peaks with coupling constants at 7.41 and 7.56 ppm, respectively. All other spectral data confirmed the structure of HT 81.

To a reaction mixture of 3,4-dimethoxy acetophenone (0.41 g, 2.27 mmol) and 3-methoxy-4-((3-methylbut-2-en-1-yl)oxy)benzaldehyde (0.50 g, 2.27 mmol) in EtOH (10mL) was added 50% NaOH (0.73 mL, 9.08 mmol). The reaction mixture was stirred at room temperature (24 h) and water was added. After extracting the mixture with ethyl acetate, organic layer was collected, washed with water and dried over anhydrous MgSO4. Solvent was removed under reduced pressure and the residue was purified by silica gel column chromatography (eluent: ethyl acetate : *n*-hexane = 1:3) to give compound **HT81** (0.71 g, 81.6%) as a yellow solid. R_f 0.53 (ethyl acetate : n-hexane = 1:1); m.p. 95 - 96 °C; HPLC: R_T 8.17 min (purity; 99.9%); ^{1}H-NMR (400 MHz, $CDCl_3$) δ 1.75 (s, 3H), 1.78 (s, 3H), 3.94 (s, 3H), 3.96 (s, 3H), 3.97 (s, 3H), 4.64 (d, *J* = 5.6 Hz, 2H), 5.50 ~ 5.54 (m, 1H), 6.90 (d, *J* = 8.4 Hz, 1H), 6.93 (d, *J* = 8.8 Hz, 1H), 7.16 (d, *J* = 2.0 Hz, 1H), 7.22 (dd, *J* = 8.4, 2.0 Hz, 1H), 7.41 (d, *J* = 15.6 Hz, 1H), 7.62 (d, *J* = 2.0 Hz, 1H), 7.67 (dd, *J* = 8.4, 2.0 Hz, 1H), 7.76 (d, *J* = 15.6 Hz, 1H); ^{13}C-NMR (100 MHz, $CDCl_3$) 18.5, 26.1, 56.2, 56.3, 66.0, 110.2. 110.7, 11.1, 112.9, 119.6, 119.8, 122.9, 123.0, 128.1, 131.8, 138.4, 144.5, 149.5, 149.8, 150.9, 153.0, 188.9 ppm; HRMS-ESI (m/z) $[M+H]^+$ $C_{23}H_{27}O_5$ calcd 383.1853, found 383.1852.

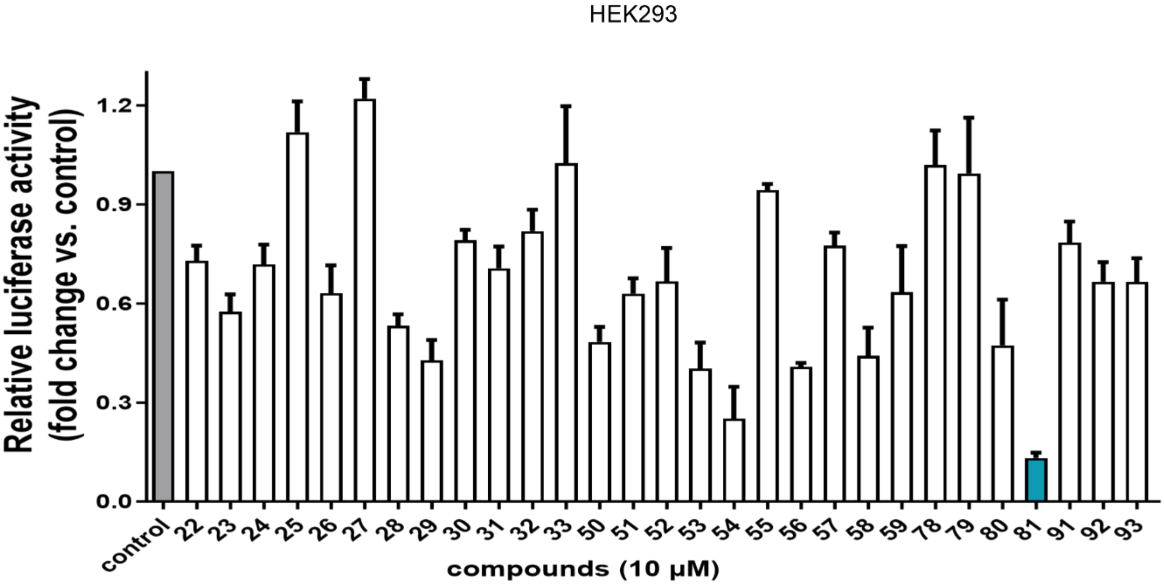

Supplementary Figure S1. Screening of chalcone-derived compounds for inhibition of the ELF3–HSP27 interaction.

Split luciferase-based biosensor assay used to evaluate the inhibitory effects of 29 chalcone-derived compounds on the ELF3–HSP27 interaction in HEK293 cells. Relative luminescence signals were normalized to the control group. HT81 showed the strongest inhibitory effect among the tested compounds. Data are presented as mean ± SD (n = 3 independent experiments).

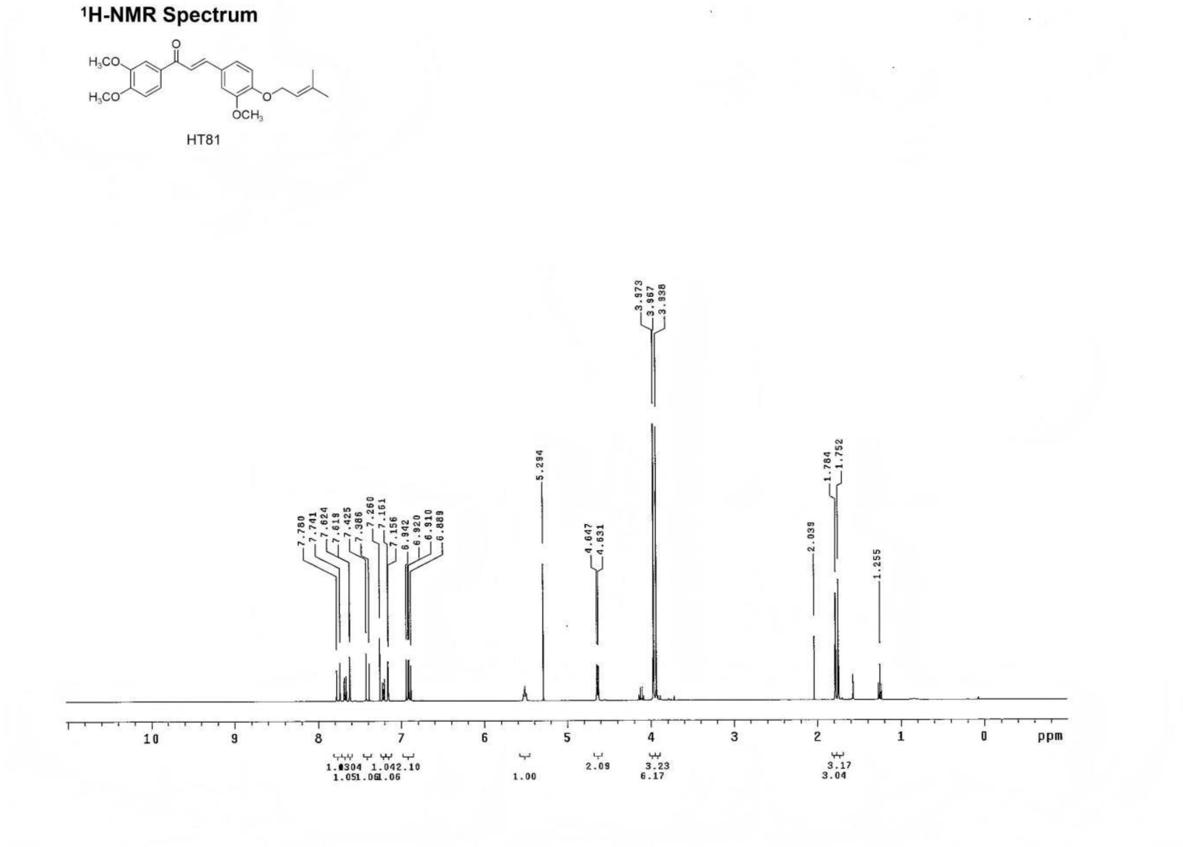

Figure S2. ^{1}H-NMR Spectrum of **HT81**

^{13}C-NMR Spectrum

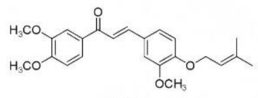

HT81

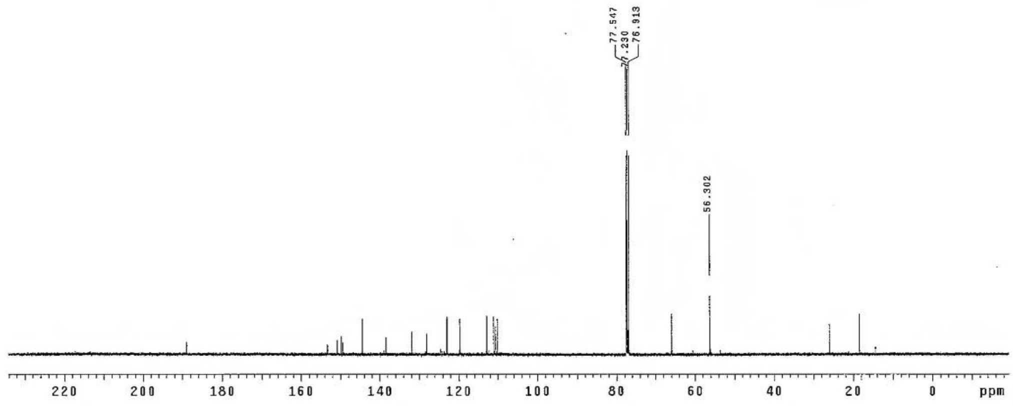

Figure S3. ^{13}C-NMR Spectrum of **HT81**

Table S1. PCR primer sequences used in this study

Gene	Forward primer (5′ → 3′)	Reverse primer (3′ → 5′)
HSPB1	CCTGGATGTCAACAACTTCG	CTGGGATGGTGATCTCGTTG
ELF3	GTGATGCTGAGCTTGGGATG	TTAGGTTAGAAGCGCCCACA
GAPDH	GAGTCAACGGATTTGGTCGT	GACAAGCTTCCCGTTCTCAG

Table S2. Information on antibodies used in this study.

Antibody	Vendor	Cat No.	Dilution rate for western blot	Dilution rate for IF
ELF3	Genetex	GTX100250	1:2000	1:200
HSP27	Santa cruz	Sc-13132	1:1000	1:200
GAPDH	MBL	M171-3	1:5000	
Vinculin	MBL	PM088	1:4000	
FLAG	MBL	M185-3L	1:2000	
c-PARP	Cell signaling	9541s	1:2000	
Survivin	Cell signaling	2803s	1:1000	1:200
α-tubulin	MBL	M175-3	1:5000	
GST	MBL	M209-3	1:2000	
Ki67	Dako	M7240		1:100
Alexa Fluor 488 anti-mouse IgG Fab2	Cell signaling	4408s		1:100
Alexa Fluor® 568 goat anti-rabbit (H+L)	Thermo Fisher Sccientific	1698376		1:100
